# Diff-AMP: tailored designed antimicrobial peptide framework with all-in-one generation, identification, prediction and optimization

**DOI:** 10.1093/bib/bbae078

**Published:** 2024-03-05

**Authors:** Rui Wang, Tao Wang, Linlin Zhuo, Jinhang Wei, Xiangzheng Fu, Quan Zou, Xiaojun Yao

**Affiliations:** School of Data Science and Artificial Intelligence, Wenzhou University of Technology, 325000 Wenzhou, China; School of Data Science and Artificial Intelligence, Wenzhou University of Technology, 325000 Wenzhou, China; School of Data Science and Artificial Intelligence, Wenzhou University of Technology, 325000 Wenzhou, China; School of Data Science and Artificial Intelligence, Wenzhou University of Technology, 325000 Wenzhou, China; College of Computer Science and Electronic Engineering, Hunan University, 410012 Changsha, China; Institute of Fundamental and Frontier Sciences, University of Electronic Science and Technology of China, 611730 Chengdu, China; Faculty of Applied Sciences, Macao Polytechnic University, 999078 Macao, China

**Keywords:** antimicrobial peptides (AMPs), AMP generation, integrated framework, diffusion, attention mechanisms, reinforcement learning

## Abstract

Antimicrobial peptides (AMPs), short peptides with diverse functions, effectively target and combat various organisms. The widespread misuse of chemical antibiotics has led to increasing microbial resistance. Due to their low drug resistance and toxicity, AMPs are considered promising substitutes for traditional antibiotics. While existing deep learning technology enhances AMP generation, it also presents certain challenges. Firstly, AMP generation overlooks the complex interdependencies among amino acids. Secondly, current models fail to integrate crucial tasks like screening, attribute prediction and iterative optimization. Consequently, we develop a integrated deep learning framework, Diff-AMP, that automates AMP generation, identification, attribute prediction and iterative optimization. We innovatively integrate kinetic diffusion and attention mechanisms into the reinforcement learning framework for efficient AMP generation. Additionally, our prediction module incorporates pre-training and transfer learning strategies for precise AMP identification and screening. We employ a convolutional neural network for multi-attribute prediction and a reinforcement learning-based iterative optimization strategy to produce diverse AMPs. This framework automates molecule generation, screening, attribute prediction and optimization, thereby advancing AMP research. We have also deployed Diff-AMP on a web server, with code, data and server details available in the Data Availability section.

## INTRODUCTION

The rise of multi-drug-resistant *superbugs* has heightened the urgent need for novel antibacterial drugs, although research and development face challenges like lengthy development cycles and drug resistance [1, 2]. Antimicrobial peptides (AMPs) are known for their low drug resistance and toxicity, have garnered significant attention as potential alternatives to antibiotics. Discovered in the 1980s in insects and animals, AMPs have been recognized as highly promising in addressing antibiotic resistance [3]. AMPs are prevalent in nature and key to the innate immune system, demonstrate broad-spectrum activity against bacteria, fungi and cancer cells [4]. In contrast to antibiotics, AMPs typically target structures like bacterial membranes, significantly reducing the risk of microbial resistance [5].

Historically, discovering new AMPs mainly involved finding active peptides from natural sources [6]. This led to creating synthetic peptides and analyzing their structure and function. Novel design methods for peptides include extensive library screening, structure-based modeling and making unique modifications [7]. Searching protein sequences also helps find peptides with antibacterial potential [8]. Some proteins naturally have antibacterial properties, increasing the variety of AMPs [9]. However, designing, screening and optimizing AMPs face challenges like high toxicity [10], sensitivity to extreme environments [11, 12], specificity issues, folding problems in larger AMPs [13], microbial resistance [2, 14] and high production costs [15]. The rise of deep learning in protein prediction [16] has enhanced AMP research in terms of creation, identification, prediction and improvement.

Deep learning models that combine peptide sequence data and biophysical assay results have improved the generation of new AMPs. For example, Generative Adversarial Network (GAN) [17] and Variational Autoencoder (VAE) [18] have made AMP generation more efficient, especially against pathogenic microorganisms, with broad applications in medicine and biology [19, 20]. Ma *et al*. used a deep neural network (DNN) to generate AMPs from human intestinal microbiome data, finding many biologically active AMPs [21]. Colin *et al*. introduced AMPGAN v2, a bidirectional conditional GAN for AMP generation, marking significant progress [22]. This method combines various networks to learn from past AMP data and uses an Encoder for repeated training, aiding in generating and identifying AMP sequences important for drug and antiviral research. Building on this, Tucs *et al*. developed PepGAN [23], specifically to generate highly active AMPs, outperforming traditional ampicillin in antibacterial activity. This is a significant step forward in developing new antibiotics against drug-resistant strains. Das *et al*. introduced a VAE-based model for creating new AMP sequences from a learned latent space, providing flexibility in custom AMP generation [24]. However, these models often do not account for the stability of the AMP’s secondary structure, which is vital for antibacterial activity [25].

In AMP research, deep learning plays a pivotal role, especially in the identification and screening of AMPs. Most models employ graph neural networks (GNN), convolutional neural networks (CNN), Transformers for predicting AMP activity [26–28]. Initially, researchers used traditional machine learning methods for this, like the Target-AMP classifier developed by Jan *et al*., which combined various feature extraction methods [29–32]. However, machine learning has limitations, such as relying on manually extracted features. Deep learning overcomes these limitations, improving AMP identification and screening. For example, Veltri *et al*. developed a DNN-based classifier for better AMP recognition [33]. Following this, various DNN models have been created to tackle AMP identification challenges. Colin *et al*. proposed sAMP-PFPDeep, a DNN-based model that uses image data and image models like ResNet-50 [34] and VGG-16 [35] for identifying potential AMPs [36]. Yan *et al*. introduced sAMPpredGAT, a deep GNN-based model, integrating peptide structure, evolutionary profiles and sequence features to identify potential AMPs using a graph attention network (GAT) [37, 38]. These advancements show that using diverse information sources increases AMP recognition accuracy.

Additionally, deep learning is increasingly used for predicting molecular attributes, including those of MPs [39–43]. For example, Xiao *et al*. created iAMP-CA2L [44], a model that uses deep learning to predict AMPs and their functions. It transforms peptide sequences into cellular automata images using ANTIALIAS [45, 46], then uses a combination of CNN, BILSTM and SVM to distinguish AMPs from non-AMPs and annotate up to 10 different AMP activities. Meng *et al*. developed multi-AMP [47], based on CNN and residual modules, which identifies AMPs and performs functional annotation in a multi-task framework. Both models use CNN for feature extraction but have different training methods for AMP identification and function prediction. While effective in function annotation, these models are limited in predicting multiple attributes, suggesting that expanding their prediction capabilities is a valuable future direction.

Recently, Lin *et al*. introduced ESM-2 [48], a pre-trained protein language model. Its exceptional generalization capability has led to its widespread use in protein sequence-based deep learning tasks. The model supports various downstream tasks, such as sequence classification, by learning general features from millions of protein sequences. Inspired by this, we plan to integrate this approach into AMP’s multi-attribute prediction tasks.

Nevertheless, several challenges persist in the implementation of AMP generation. Firstly, existing AMP generation models overlook potential dependencies between nodes, hindering efficient generation processes. Secondly, current AMP screening models struggle to extract general features, leading to challenges in accurately identifying potential AMPs. Thirdly, the omission of attribute prediction in existing AMP generation models complicates further screening. Fourthly, current models lack the capability to customize AMPs according to user-specified attributes. To address these issues, we propose a comprehensive framework that integrates AMP generation, screening, prediction and optimization, utilizing diffusion, pre-training and iterative optimization technologies to advance AMP research. Our primary contributions are as follows:

(i) We develop Diff-AMP, a integrated framework for AMP generation, integrating functions such as identification, prediction and optimization to enhance the efficiency of AMP development and research.
(ii) We design RLGen, an AMP generation model employing a diffusion strategy to enhance the interdependence between amino acid nodes, thus enabling efficient AMP generation.
(iii) We incorporate a high-parameter pre-training model to develop the AMP recognition model, enhancing node representation learning and improving screening efficiency.
(iv) Utilizing a reinforcement learning approach, we train an AMP iterative optimization model to generate AMPs with specific attributes, catering to user-defined requirements like certain attributes and length ($\geq $ 6 amino acids).

## METHODS

We develop Diff-AMP, a integrated framework for AMP generation and optimization, combining various deep learning technologies to efficiently accomplish AMP generation, evaluation and optimization tasks.

### Model architecture

**Figure 1:**
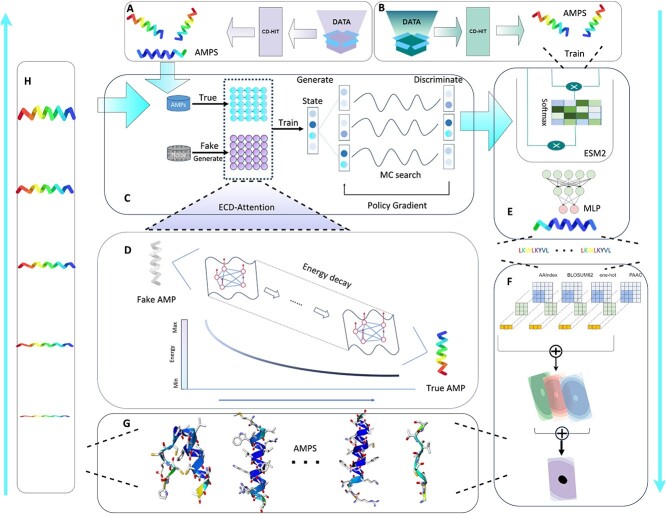
The overall architecture of the Diff-AMP framework, which mainly includes four modules: Data Preparation (**A**) and (**B**), AMP generation (**C**), recognition (**E**), attribute prediction (**F**) and iterative optimization (H). C represents the AMP generation module, including ECD-Attention encoder (**D**), generator and discriminator. E represents the AMP recognition module, which infers whether the generated sequences are potential AMPs. F represents the AMP multi-attribute classification module, which performs multi-attribute prediction on the generated AMPs. H represents the iterative optimization module, which selects specific attributes for iterative training based on reinforcement learning strategies, and then focuses on generating AMPs that meet certain attributes. And MC search refers to Monte Carlo search.

As illustrated in Figure 1, this framework executes a comprehensive system of AMP generation, judgment, attribute prediction and multi-objective iterative optimization for automated molecular design and optimization. This framework comprises four modules, each utilizing advanced deep learning technologies. In the first round of iteration, in module A, the original AMP data are filtered by CD-HIT to form the dataset required by AMP generation module C. In module B, the original AMP data is filtered by CD-HIT (http://cd-hit.org) to form the dataset required by AMP recognition module E. In module C, we integrate the attention mechanism (D) based on the energy diffusion process into the GAN network for AMP generation. Module E introduces a high-parameter pre-trained model that can learn general knowledge of AMPs and efficiently infer potential AMPs. Module F uses multiple CNN models to perform the AMP multi-attribute prediction task and retains AMPs (G) that meet the conditions. Module H uses reinforcement learning strategies to continuously and iteratively optimize the generated AMPs. In round 2 and subsequent iterations, the Diff-AMP framework executes the C-E-F-G-H-C workflow. The framework aims to generate AMPs that meet user-specified attributes and improve the quality and diversity of generated AMPs. Additionally, we provide free access to our code and online servers in the Data Availability section. Next, we detail the underlying technologies and principles.

### AMP generation and design

In this subsection, we introduce RLGen, a novel AMP generation model that integrates the thermodynamic diffusion model [49] with reinforcement learning [50]. The model’s attention mechanism, informed by the thermodynamic diffusion process, effectively captures the interdependence between amino acids and enhances the characterization of information propagation during node representation updates. This approach allows for a more accurate simulation of AMP conformation and amino acid interactions. To our knowledge, this represents the first integration of thermodynamic diffusion and attention mechanisms into a reinforcement learning framework. The goal is to produce AMP candidates that are both highly diverse and biologically active.

**Figure 2:**
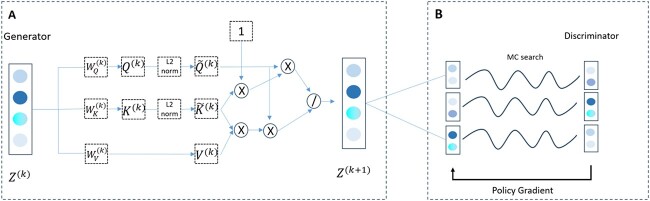
The overall architecture of the RLGen model, which mainly includes two modules: AMP generator (**A**), discriminator (**B**), where MC search refers to Monte Carlo search.

The RLGen model, depicted as module A in Figure 2, comprises two main components: a generator and a discriminator. The generator’s primary function is to create fictitious AMP sequences, whereas the discriminator distinguishes real AMPs from the generated ones. Figure 2 illustrates the detailed workflow of the RLGen model, with Figure 2(A) depicting the generator and Figure 2(B) demonstrating the iterative training of the generator and discriminator based on reinforcement learning. The fully trained RLGen model will ultimately be utilized to generate novel AMP candidates.

#### Energy-constrained diffusion process

The generator, a crucial component of the RLGen model, is based on an attention mechanism driven by the thermodynamic diffusion process. In subsequent discussions, these nodes will be referred to as amino acids in the peptide sequence. To further elucidate the generator’s design rationale, we introduce the energy-constrained diffusion process equation: 


(1) \begin{align*} & \begin{aligned} & h_{i}^{l+1}=(1-\alpha \sum_{j=1}^{N}S_{ij}^{l})h_{i}^{l}+\alpha \sum_{j=1}^{N}S_{ij}^{l} h_{j}^{l},\\ & s.t. \quad h_{i}^{0}=x_{i}, \; E(H^{l+1},l+1,\vartheta)\leq E(H^{l},l,\vartheta),\; l\geq 1, \end{aligned}\end{align*}

where 


(2) \begin{align*} & E(H^{l},l,\vartheta)=\Arrowvert H-H^{l} \Arrowvert + \beta \sum_{i,j}\vartheta(\Arrowvert h_{i}-h_{j} \Arrowvert^{2}_{2}).\end{align*}

In the above equations, $h_{i}^{l}$ denotes the energy state of node $i$ at the $l$th moment, while $S_{ij}$ defines the diffusion rate, indicative of the rate at which energy propagates. $\alpha $ represents the weight factor regulating the node’s energy change and the energy transfer to other nodes. Equations (1) and (2) describe the diffusion equation under energy constraints. Equation (1) specifies the node’s energy state update equation, where the first segment of constraints sets the initial constraint condition, and its second segment outlines the energy reduction constraint condition. Equation (2) denotes the energy function that steers the energy propagation in the diffusion process via energy minimization. Additionally, Equation (3) embodies the potential consistency within the node-system. Its first term indicates the node’s own energy propagation consistency, while the second term signifies the system-wide energy propagation consistency among the node and all other nodes.

Naturally, optimizing this equation presents a significant challenge. This is due to the need to compute a series of diffusivity values that meet specific conditions under various constraints. Consequently, we propose a feasible solution for determining the $S^{l}$ value. Given that the weight $\alpha $ must fall within the (0,1) interval, an estimation equation for the diffusion rate is as follows: 


(3) \begin{align*} & \hat{S}_{ij}^{l}=\frac{w_{ij}^{l}}{\sum_{k=1}^{N} w_{ik}^{l}},\; w_{ij}^{l}=\frac{\partial \vartheta(h^{2})} {\partial h^{2}}, \; h^{2}=\Arrowvert h_{i}^{l}-h_{j}^{l} \Arrowvert^{2}_{2},\end{align*}

where $w_{ij}^{l}$ represents the similarity between node $i$ and node $j$ in the $l$th state. Clearly, Equation (3) can be considered as the calculation of attention between any two nodes at this stage. Building upon this theory, we can further derive the Transformer structure that guides the diffusion process: 


(4) \begin{align*} & \hat{S}_{ij}^{l}=\frac{f(\Arrowvert h_{i}^{l}-h_{j}^{l} \Arrowvert^{2}_{2})}{\sum_{k=1}^{N} f(\Arrowvert h_{i}^{l}-h_{k}^{l} \Arrowvert^{2}_{2})},\end{align*}

In this structure, $f$ denotes the similarity measure function. To ensure the non-negativity of the $f$ function’s value, assume $\hat{h}_{i}^{l}=\frac{h_{i}^{l}}{\Arrowvert h_{i}^{l} \Arrowvert _{2}}$, $\hat{h}_{j}^{l}=\frac{h_{j}^{l}}{\Arrowvert h_{j}^{l} \Arrowvert _{2}}$ and select the linear function $f(x)=2-1/2x$. Consequently, $w_{ij}^{l}$ can be further expressed as follows: 


(5) \begin{align*} & w_{ij}^{l}=f(\Arrowvert \hat{h}_{i}^{l}-\hat{h}_{j}^{l} \Arrowvert^{2}_{2})=1+(\frac{h_{i}^{l}}{\Arrowvert h_{i}^{l} \Arrowvert_{2}})^{\top} (\frac{h_{j}^{l}}{\Arrowvert h_{j}^{l} \Arrowvert_{2}}).\end{align*}

Finally, $w_{ij}^{l}$ is integrated into the aggregation equation for a single node: 


(6) \begin{align*} & \begin{aligned} &\sum_{j=1}^{N}S_{ij}^{l} h_{j}^{l}=\sum_{j=1}^{N}\frac{1+(\hat{h}_{i}^{l})^{\top} \hat{h}_{j}^{l}}{\sum_{k=1}^{N} (\hat{h}_{i}^{l})^{\top} \hat{h}_{k}^{l}} h_{j}^{l} \\ & = \frac{\sum_{j=1}^{N} h_{j}^{l} + (\sum_{j=1}^{N} \hat{h}_{j}^{l} \cdot (h_{j}^{l})^{\top} ) \cdot \hat{h}_{i}^{l}}{N+(\hat{h}_{i}^{l})^{\top} \sum_{k=1}^{N} \hat{h}_{k}^{l}} \end{aligned}\end{align*}

And the calculation process is depicted in the ECD-Attention module of Figure 2(A).

#### Transformer with ECD-Attention mechanism

In the ECD-Attention mechanism, the attention weight between two amino acids can be regarded as the diffusion rate between nodes. Inspired by the previous work [49], the ECD-Attention mechanism is integrated into the Transformer architecture. According to Equation 6, the Transformer architecture can be designed as shown in Figure 2(A).

First, the learned sequence feature $X$ of AMPs is input into the linear layer for layer normalization operation: 


(7) \begin{align*} & H=\sigma(\delta(WX+b)),\end{align*}

where $\sigma $ represents the activation function, $\delta $ represents the layer normalization operation, $W$ represents the linear layer parameters and $b$ represents the bias.

Then, linear layers are used to calculate the $Q$, $K$ and $V$ matrices separately: 


(8) \begin{align*} & K^{(k)}=W^{(k)}_{K}H^{(k)}, \; Q^{(k)}=W^{(k)}_{Q}H^{(k)}, \; V^{(k)}=W^{(k)}_{V}H^{(k)},\end{align*}

where $K^{(k)}$, $K^{(k)}$ and $K^{(k)}$ represent the $Q$, $K$ and $V$matrices of the $k$th layer, respectively, and $W^{(k)}_{K}$, $W^{(k)}_{Q}$ and $W^{(k)}_{V}$ are their corresponding linear layer parameter matrices.

Immediately, the $K$ and $Q$ matrices are normalized using the $L_{2}$ norm: 


(9) \begin{align*} & \tilde{K}^{(k)}=\left[ \frac{K_{i}^{(k)}}{\Arrowvert K_{i}^{(k)} \Arrowvert_{2}} \right]^{N}_{i=1}, \; \tilde{Q}^{(k)}=\left[ \frac{Q_{i}^{(k)}}{\Arrowvert Q_{i}^{(k)} \Arrowvert_{2}} \right]^{N}_{i=1}.\end{align*}

Finally, as shown in Figure 2(A), after multiple operations on the $K$, $Q$ and $V$ matrices, the embedding of the $k+1$th layer is obtained.

Unlike GRU and LSTM, the proposed Transformer considers both the evolutionary direction of individual amino acids in the peptide sequence and the global evolutionary consistency between any two amino acids. This helps to uncover the intricate interdependencies among amino acids. Additionally, global consistency constraints introduced in this module help mitigate the impact of noise. Consequently, the generator is capable of producing a range of diverse and biologically active AMP candidates.

In summary, our AMP generation model, leveraging diffusion and attention mechanisms along with reinforcement learning strategies, is anticipated to significantly enhance AMP generation quality. Furthermore, we trained the proposed RLGen model using a benchmark dataset [51] comprising 8225 AMP sequences sourced from the CAMP server [52]. Accomplishing this process requires substantial computing resources. Following a series of optimization measures, it is now feasible to train user-customized AMP generation models with specific attributes, even on devices with limited memory capacity.

### AMP identification

To identify the most promising AMPs from the generated candidates, we utilize transfer learning and the pre-trained ESM2 model [48]. ESM2 is a model pre-trained on extensive protein data, can learn universal features from an immense dataset encompassing tens of billions of proteins. The ESM2 model is adaptable to our AMP data, thus improving the identification model’s generalization performance.

For downstream AMP classification, we employ a multi-layer perceptron (MLP) for feature learning, predicting AMP performance up to the final selection. This approach aids in identifying the most promising candidate molecules.

### AMP attribute prediction

For the AMP attribute prediction task, we implement a CNN model utilizing multi-hybrid embedding, detailed in Module D of Figure 1. Prior research [53] has demonstrated the efficacy of this model in predicting AMP attributes. Specifically, we encode the AMP sequence using four methods—one-hot, BLOSUM62, AAIndex and PAAC—each subsequently fed into the convolutional neural network. Subsequently, a specialized fusion technique is applied to these four embeddings, with the resultant fused features input into the fully connected layer for attribute prediction. Integrating multi-source features extracted from AMP sequences enabled the accurate prediction of several key AMP attributes, including biological activity, antibacterial and anti-cancer properties.

However, multi-layer embedding significantly increases the consumption of computing resources and time. To address this issue, we employed mixed precision training technology [54]. Mixed precision training accelerates the process by reducing the number of floating-point digits, trading off some accuracy for a substantial reduction in training time. To balance model performance and accuracy, we also integrated R-Drop [55] regularization technology. This technique involves double forward propagation, or double dropout, to generate two closely related but slightly different probability distributions. Subsequently, the symmetric KL divergence loss of these distributions is added to the original cross-entropy loss for backpropagation and parameter updating. This approach addresses inconsistencies in training and inference predictions due to Dropout, thereby enhancing the model’s expressiveness and generalizability.

### AMP multi-attribute iterative optimization

We employ a multi-objective iterative optimization strategy, in conjunction with reinforcement learning, to continuously refine the generated candidate AMPs. Initially, AMP sequences meeting one or more specific attributes are collected; these are then input into the RLGen model for iterative optimization and regeneration. This process notably enhances the prominence of specific attributes. Consequently, the optimal solution is sought within a multi-dimensional target space to generate AMPs fulfilling specific attributes. The goal of this multi-attribute iterative optimization is to enhance AMP’s biological activity and reduce toxicity, among other improvements.

### Custom attribute generation

Ultimately, we developed a one-click model for generating AMPs with selected specific attributes. Researchers can easily customize AMPs using our model by simply adjusting parameters to generate AMPs with desired attributes. Additionally, researchers have the option to modify data sources, like protein sequences and train models from scratch. This capability extends to generating custom sequences, including macromolecular peptides, beyond just short peptides like AMPs. The model has undergone multiple optimizations, enabling its use for training from scratch even by institutions or individuals with limited computing resources.

## RESULTS

### Datasets

**Figure 3:**
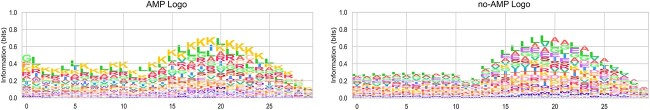
Distribution of positive and negative samples in the dataset. We use blank characters for padding to ensure that this does not affect the analysis of the relative positions of the AMP sequences. In no-AMP sequences, specific L sites have a higher frequency. In the AMP sequence, specific K sites have a higher frequency.

Our dataset comprises three main parts: dataset1 [51], dataset2 [51] and dataset3 [53]. Dataset1, sourced from the CAMP server [45], includes 8225 AMP sequences primarily utilized for AMP generation and iterative optimization training. Dataset2 comprises 8268 samples, evenly split with 4134 AMP and 4134 non-AMP samples. Figure 3 displays a sequence annotation diagram for positive and negative samples in dataset2, illustrating the conservation and information content across various sequence positions. Figure 3 reveals a distinct positional preference difference between positive and negative AMP samples. AMP sequences typically show a higher frequency of K sites, whereas L sites are more prominent in non-AMP sequences. This difference, possibly due to their belonging to distinct families, may aid in training AMP recognition models.

Dataset3, named after the work referenced in [53], serves as training data for AMP multi-attribute prediction. This dataset includes 20 types of AMPs, each with distinct properties such as antibacterial, anti-Gram-positive, anti-Gram-negative, antifungal, antiviral, anti-mammalian-cells, anti-HIV, antibiofilm, anti-methicillin-resistant Staphylococcus aureus (MRSA), antiparasitic, hemolytic, chemotactic, anti-TB, anurandefense, cytotoxic, endotoxin, insecticidal, antimalarial, anticandida and antiprotozoal. All data are utilized in this study, with detailed statistics presented in Tables 1 and 2.

**Table 1:** Detailed statistics of dataset1 and dataset2.

Datasets	Positive	Negative
dataset1	8225	-
dataset2	4134	4134

**Table 2:** Detailed statistics of dataset3.

Attribute datasets	Positive	Negative
Antibacterial	16 058	13 551
Antibiofilm	372	29 202
Anticancer	4698	24 876
Anticandidal	669	28 905
Antifungal	5955	23 619
Anti-Gram negative	8537	21 044
Anti-Gram positive	8053	21 539
Anti-HIV	812	28 762
Antimalarial	73	29 501
Anti-MRSA	267	29 307
Antiparasitic	457	29 117
Antiplasmodial	63	29 511
Antiprotozoal	53	29 521
Anti-TB	275	29 299
Antiviral	6495	23 093
Anti-mammalian cells	4345	25 229
Anuran defense	779	28 795
Chemotactic	85	29 489
Cytotoxic	180	29 394
Endotoxin	82	29 492
Hemolytic	2383	27 191
Insecticidal	415	29 159

### Evaluation metrics

We evaluate the model’s performance using metrics such as ACC, PRE, SEN, SPE, MCC, F1-Score, AUC and IDDT. The calculations for these metrics are as follows: 


(10) \begin{align*} & ACC=\frac{TP + TN}{TP + TN + FP + FN},\; SPE=\frac{TN }{TN + FP}, \end{align*}

(11) \begin{align*} & PRE=\frac{TP}{TP + FP},\; SEN=\frac{TP }{TP + FN},\; \end{align*}

(12) \begin{align*} & F1-Score=\frac{2 PRE\cdot SEN}{PRE+SEN}, \end{align*}

(13) \begin{align*} & MCC=\frac{TP\cdot TN - FP\cdot FN}{\sqrt{(TP + FP)(TP + FN)(TN + FP)(TN + FN)}} \end{align*}

where TP is the positive samples correctly identified, TN is the negative samples correctly identified, FP is the negative samples incorrectly labeled and FN is the positive samples incorrectly labeled.

AUC refers to the *area under the ROC (Receiver Operating Characteristic) curve*, commonly used to assess the performance of binary classification models, such as machine learning classifiers. The ROC curve illustrates the relationship between the true positive rate and the false positive rate at various classification thresholds. IDDT assesses the structural similarity between two proteins. In our experiments, we compared the predicted AMP structure with experimentally verified AMP structures, calculating similarity at each position based on spatial distribution and atomic deviation to assess the prediction model’s confidence performance.

### Experimental results

In this section, we evaluate our framework’s performance focusing on AMP recognition efficiency, AMP generation quality and AMP attribute prediction efficiency. Additionally, we investigate the effects of pre-trained models and model parameters on our framework. Furthermore, we optimized the model to balance accuracy and computational speed.

#### Comparison of AMP recognition performance

We compared our AMP recognition model’s performance with four state-of-the-art models: Cao *et al*. [51], Amplify [56], AMPScannerV2 [33], MACREL [57] and AmPEPpy [58] using a benchmark dataset. And the results are presented in Table 3. Overall, both the Cao *et al*.’s model and our proposed model, which employs a pre-training strategy, outperform models without pre-training. This advantage stems from the pre-training strategy’s ability to learn general knowledge from extensive data. Although models like AMPlify and AMPScannerV2 utilize various deep learning technologies, including Bi-LSTM and convolutional neural networks, they struggle to extract general knowledge from AMP sequences. Consequently, large-scale pre-training followed by task-specific fine-tuning emerges as a reliable approach for AMP recognition models.

**Table 3:** Performance comparison of multiple AMP recognition models (%).

Models	ACC	PRE	SEN	SPE	MCC	F1-Score	AUC
AmPEPpy	79.99	87.01	70.50	89.48	61.09	77.38	87.41
MACREL	82.35	85.43	77.99	86.70	64.94	81.60	89.32
AMPScannerV2	82.83	85.87	78.60	87.06	65.90	82.12	90.10
AMPlify	84.16	88.97	77.89	90.33	68.84	83.07	90.69
Cao *et al*.	85.31	87.92	81.86	88.75	70.79	84.71	91.41
Our model	86.92	91.48	83.02	91.44	74.07	86.39	92.22

Notably, all evaluation metrics of our proposed model surpass those of the other models. The proposed model’s ACC, PRE, SEN, SPE, MCC, F1-Score and AUC are, respectively, 1.61, 3.56, 1.16, 2.69, 3.28, 1.68, 2.76% and 0.8% higher than the second-best Cao *et al*.’s model, and 2.51, 5.13, 1.11, 5.23, 3.12% and 1.52% higher than the third-ranked model. Table 4 shows that the 10-fold cross-validation results indicate minimal impact of data partitioning on model performance. This evidence confirms the proposed AMP identification model’s efficiency in discovering potential AMPs. The proposed model’s selection of an appropriate pre-training model achieves a balance between performance and efficiency. This further illustrates that the parameters and training data of the pre-trained model significantly influence its ability to fit downstream data.

**Table 4:** Results of 10-fold cross-validation of the proposed AMP recognition model on dataset2 (%).

Round	ACC	PRE	SEN	SPE	MCC	F1-Score	AUC
1	86.54	91.32	83.27	91.45	73.89	86.12	92.08
2	87.32	91.78	82.58	91.93	74.55	86.70	92.37
3	86.58	91.21	83.45	91.01	73.67	86.21	92.15
4	87.01	91.63	82.95	91.36	74.32	86.51	92.25
5	86.93	91.42	83.12	91.27	73.98	86.29	92.21
6	87.05	91.75	82.71	91.60	74.44	86.63	92.32
7	86.79	91.06	83.64	90.84	73.45	86.06	92.03
8	87.13	91.89	82.42	92.01	74.68	86.83	92.42
9	86.95	91.29	83.00	91.49	73.79	86.24	92.18
10	86.92	91.47	83.12	91.49	74.00	86.33	92.22
Average	86.92	91.48	83.02	91.44	74.07	86.39	92.22

#### Comparison with sSub-optimal models

**Figure 4:**
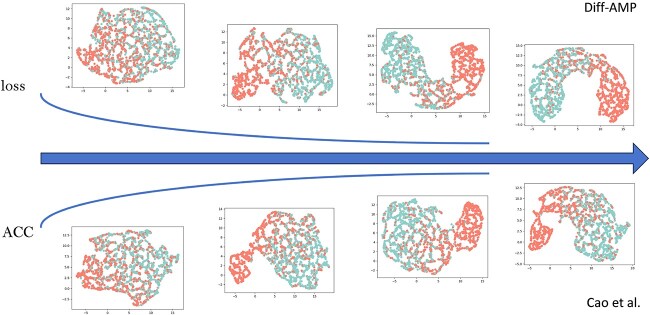
Comparison of our designed AMP recognition model with sub-optimal models. The UMAP method is used to obtain the diagrams.

We employ Uniform Manifold Approximation and Projection (UMAP) technology [59] to intuitively compare the performance of our proposed model with sub-optimal models in AMP recognition. UMAP, a widely-used visualization tool, reveals data’s fundamental characteristics via dimensionality reduction and visualizes the model’s feature output spatial distribution. Figure 4 presents the visualization comparison between the proposed model and the sub-optimal model.

In Figure 4, red dots represent AMP samples, while blue dots denote non-AMP samples. During model training, the loss functions of both models gradually decrease, leading to improved classification effects. Notably, the proposed model demonstrates greater effectiveness in distinguishing between AMP and non-AMP samples compared to the sub-optimal model. Additionally, the sample points within each category are more uniformly and compactly distributed. This indicates that the proposed model more effectively learns feature representations from samples across different categories.

The performance improvement can be attributed to several factors: firstly, our adoption of an effective pre-trained model enables the capture of extensive contextual semantic information from millions of background genome sequences. Secondly, we fine-tune the pre-trained model to tailor it to the specific task at hand. Additionally, we utilize MLP technology to highlight crucial features, enabling a more comprehensive learning of the information embedded in the data. The synergistic integration of these technologies significantly enhances our model’s capability to analyze AMPs.

#### Comparison of different pre-trained models

Table 5 presents the outcomes of integrating various pre-trained models into our proposed model. Overall, the esm2_t12_35M_UR50D-integrated model excels in ACC, SEN, MCC, F1-Score and AUC metrics, but is less optimal in PRE and SPE. Furthermore, the esm2_t12_35M_UR50D pre-trained model has a relatively small parameter count of only 35M, contributing to reduced training time. This demonstrates that the esm2_t12_35M_UR50D-integrated model ensemble can perform AMP identification tasks both stably and efficiently.

**Table 5:** Performance comparison of the proposed model using different pre-trained models (%).

Models	ACC	PRE	SEN	SPE	MCC	F1-Score	AUC	Time	Params
esm2_t6_8M_UR50D	85.31	87.94	81.82	88.68	70.71	84.67	91.42	1	8M
esm2_t12_35M_UR50D	86.92	91.48	83.02	91.44	74.07	86.39	92.22	$\geq $ 10	35M
esm2_t30_150M_UR50D	85.59	90.14	80.01	91.20	71.66	84.81	91.43	$\geq $ 200	150M
esm2_t33_650M_UR50D	83.31	93.62	71.38	95.12	68.47	81.04	90.28	$\geq $ 5000	650M

While the esm2_t33_650M_UR50D pre-trained model excels in PRE and SPE, its performance on other metrics is significantly lower than that of the esm2_t12_35M_UR50D model. This suggests that while the large-parameter model enhances identification of negative AMPs, it compromises the detection of potential positive AMPs. Additionally, the large number of parameters increases the complexity of training. Therefore, using a pre-trained model can indeed enhance model performance. However, it is crucial to select an appropriate pre-trained model that can learn a broad spectrum of general features.

#### AMP generation quality analysis

We develop the AMP generation model RLGen, utilizing diffusion and attention mechanisms along with reinforcement learning strategies. To assess the quality of AMPs generated by RLGen, we analyze the predicted structures and various properties of these AMPs.

**Structural analysis**. To evaluate the generated AMPs’ quality, we utilized AlphaFold2 to predict the structure of these AMP sequences. The results demonstrate that our model can generate various stable AMP types, including $\alpha $-helical, $\beta $-helical and extended peptides. Notably, $\alpha $-helical peptides are the most prevalent, with extensive related research. For instance, Magainin from the African clawed frog is known for its effective antibacterial properties against E.coli and MRSA. Typically, $\alpha $-helical peptides have sufficient length to traverse double-layered cell membranes. Their structure includes $\alpha $-helices with both hydrophobic and hydrophilic regions, enabling interaction with cell membranes. This structural property facilitates their impact on bacterial membranes, potentially causing lipid dissociation, cellular content leakage or membrane depolarization. Additionally, recent studies highlight the significance of $\beta $-helical and extended peptides in understanding antibacterial mechanisms and developing new antibiotics.

**Figure 5:**
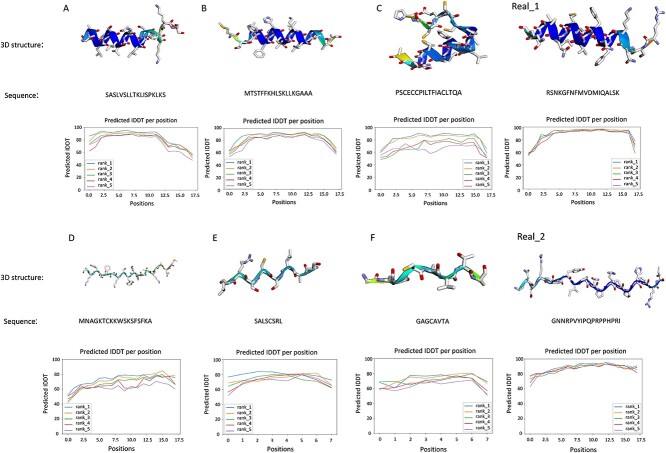
Analysis of AMP structures generated by the proposed model using AlphaFold2.0. And sequence denotes the AMP sequence, and 3D structure refers to the three-dimensional structure derived from the corresponding AMP sequence using AlphaFold2. The IDDT line chart in the figure indicates the confidence or accuracy level, calculated based on the positional similarity of each amino acid residue in the predicted AMP. These lines correspond to five distinct levels of prediction. Structures A, B, C, D, E, F, Real_1 and Real_2 represent first-level predictions, indicating they have the highest prediction values.

Figure 5 depicts structures A, B, C, D, E and F as predicted from the AMP sequences generated by our model using AlphaFold2. Real_1 and Real_2 represent structures predicted from actual AMP sequences sourced from the CAMPR4 database [52]. Clearly, A, B and Real_1 exhibit typical $\alpha $-helix arrangements; C shows a mix of $\alpha $-helices and $\beta $-sheets, while D, E and F are extended peptides. The lDDT line chart in Figure 5 serves as a metric for assessing protein structure prediction quality, indicating the confidence or accuracy of each amino acid residue’s position. Confidence scores range from 0 to 100, where high scores suggest similarity to experimentally verified structures, and low scores indicate significant differences. The line chart’s results demonstrate that the generated AMP sequences yield high-confidence structures, underscoring the efficacy of our proposed AMP generation model.

#### Multi-attribute Analysis

**Figure 6:**
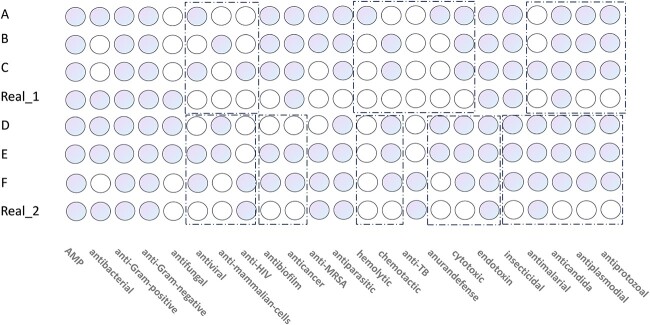
The attributes corresponding to the first-level prediction structure of AMP are generated by the proposed framework.

Peptide structures significantly influence their antimicrobial properties. We utilized our trained multi-attribute prediction model to forecast AMPs’ attributes, with results displayed in Figure 6. In Figure 6, colored circles represent the presence of a property, while uncolored circles indicate its absence. Generally, AMPs generated by our model that resemble real AMPs in structure tend to exhibit similar antibacterial properties. Conversely, structurally dissimilar AMPs often demonstrate varied antibacterial properties. Within the dotted box, it’s evident that the AMPs generated by our model possess a broader range of antibacterial properties compared to real AMPs. This could be attributed to the model undergoing several rounds of iterative training using reinforcement learning strategies, thus generating AMPs with more specific attributes.

Previous studies [60] indicate that AMPs with $\alpha $-helical structures exhibit weak antibacterial efficacy against Gram-positive bacteria, including Staphylococcus aureus. This is likely due to these bacteria’s cytoplasmic membrane composition, primarily of negatively charged lipids like phosphatidylglycerol and cardiolipin, making lipid separation challenging. Consequently, certain AMPs are less toxic to these bacteria [60]. The four AMPs A, B, C and Real_1 in the CAMPR4 database [52] that we generated exhibit resistance to Gram-positive bacteria. This demonstrates the model’s ability to learn from AMPs known for Gram-positive bacteria resistance, iteratively optimizing the generation of AMPs with this specific trait.

Generally, the length of an AMP sequence influences its antibacterial properties [61]. For instance, shorter AMP sequences, easily broken down by antibacterial enzymes, may lack membrane activity and thus exhibit weaker antibacterial properties. Conversely, longer sequences, more complex and resistant to enzymatic breakdown, tend to have stronger antibacterial effects. Notably, the shortest known active peptides, RRWQWR and RAWVAWR from bovine lactoferrin and human lysozyme, respectively, are recognized as active antibacterial fragments [7]. These peptides fold into specific amphiphilic structures in the presence of membranes, relying primarily on cytoplasmic accumulation rather than membrane activity. In Figure 6, sequences E and F are notably short, comprising only eight amino acids each. Despite their short length, E and F exhibit strong antibacterial activity, as shown in Figure 6. This could be attributed to E and F’s similarity to active peptide sequences like RRWQWR and RAWVAWR, suggesting analogous accumulation mechanisms. Moreover, the real AMP, Real_2, comprises 18 amino acids, structurally akin to D, hence exhibiting similar antibacterial properties. Furthermore, compared to the real AMP, D demonstrates enhanced antibacterial properties. In summary, the RLGen AMP generation model can produce AMPs with varying antibacterial activities, leveraging reinforcement learning strategies for sequences of different lengths. This demonstrates the model’s potential as a tool for advancing AMP-related research rapidly.

#### Multi-attribute prediction

**Figure 7:**
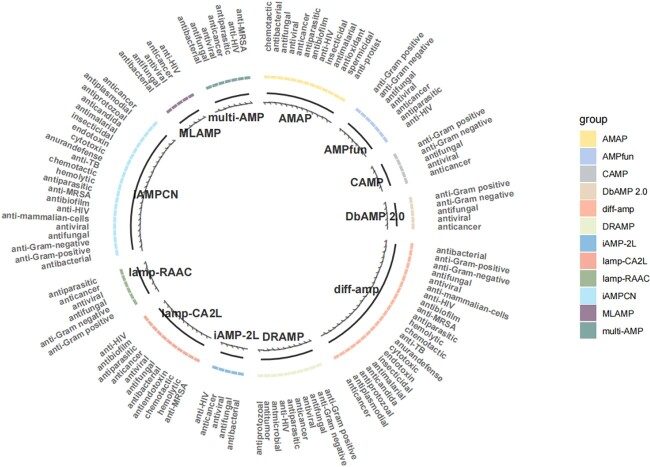
Comparison of multiple AMP attribute prediction models.

As depicted in Figure 7, numerous models currently specialize in AMP attribute prediction, ranging from initial single activity prediction to multi-attribute prediction. In our Diff-AMP framework, we have integrated leading AMP attribute prediction models, including AMAP [62], AMPfun [63], CAMP [64], DbAMP2.0 [65], DRamp [66], iamp-2l [67], iamp-CA2L [44], iamp-RACC [68], MLAMP [69], multi-AMP [47] and IAMPCN [53]. This capability allows the Diff-AMP framework to extensively predict AMP properties, encompassing over 20 attributes such as antibacterial, anti-Gram-positive, anti-Gram-negative, antifungal, antiviral and more. This enhancement significantly broadens the framework’s capacity to predict various AMP attributes.

Furthermore, we have transformed the model into a freely accessible AMP multi-attribute online prediction tool, available through a designated web address. This tool substantially reduces the resources needed for researchers to predict AMP properties, thereby advancing research and development in AMP-related fields.

#### Mixed precision + R-Drop for training

**Figure 8:**
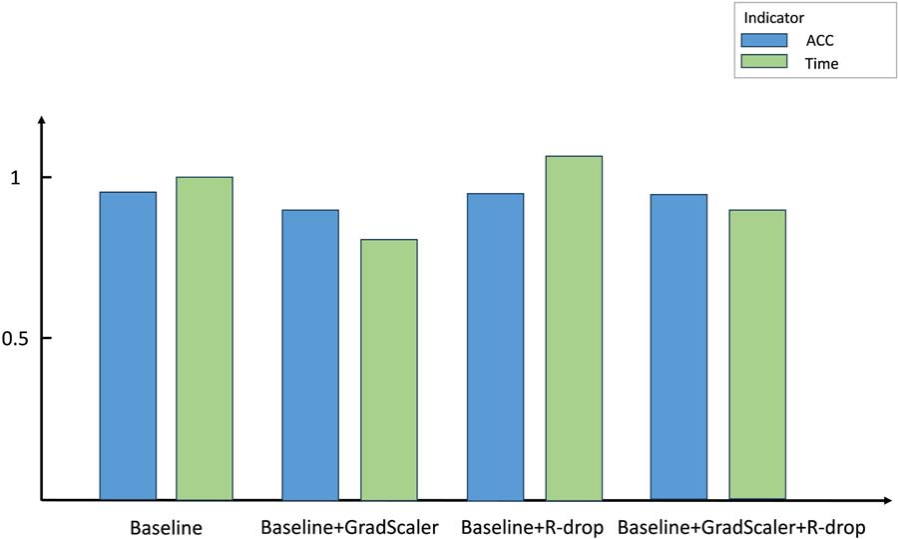
Results of various training strategies implemented on the proposed model.

We integrate mixed-precision operations and R-Drop regularization technology [70] to design a new training strategy for attribute prediction tasks. First, we use PyTorch’s GradScaler (https://pytorch.org/docs/stable/amp.html) component to automatically optimize gradients during the training process to reduce time and space overhead. At this time, it will cause a slight decrease in model accuracy. To overcome this challenge, we then introduce the R-Drop regularization technique to incorporate the loss of two forward propagations into the final optimization objective. Through this measure, the problem of decreased accuracy is alleviated. This approach balances time efficiency and model performance, offering an effective training optimization strategy. Figure 8 illustrates the performance.

## CONCLUSION

This study began with an investigation into existing research on AMPs. In a context where antibiotic use is widespread, viruses and bacteria are developing increasing resistance. AMPs, with their low toxicity and high efficiency, present a promising alternative to antibiotics. Existing deep learning methods have led to the design of various highly active AMPs, achieving significant breakthroughs. However, these methods also exhibit inherent limitations. Firstly, the interdependence between amino acids, a crucial factor, was often overlooked in AMP generation. Secondly, there is a lack of an integrated framework capable of handling tasks like AMP generation, identification and screening, multi-attribute prediction and iterative optimization. Consequently, we propose Diff-AMP, a unified framework based on various deep learning strategies and technologies, aimed at efficiently accomplishing these tasks. Furthermore, experiments and analysis have confirmed that the model excels in tasks like AMP identification and multi-attribute prediction, and is capable of synthesizing highly active AMPs.

Within the Diff-AMP framework, we have, for the first time, integrated dynamic diffusion and attention mechanisms into a reinforcement learning framework to efficiently generate AMPs. Subsequently, we incorporated pre-training and transfer learning strategies in the prediction module to enhance node representation and accurately identify and screen potential AMPs. Concurrently, we employed a multi-embedded convolutional neural network for multi-attribute prediction of the generated potential AMPs. Finally, we implemented an iterative optimization strategy based on reinforcement learning to comprehensively capture complex information, thereby generating AMPs that fulfill diverse requirements. This framework is capable of automatically completing molecule generation, screening, attribute prediction and optimization, significantly facilitating AMP research. Additionally, we have implemented these functions on an online web server. This research holds promise as a pivotal tool for generating, screening, predicting and optimizing AMPs, thereby significantly advancing the development of future antimicrobial therapies.

However, the prosed Diff-AMP framework also has some problems and fails to analyze and extract multi-modal information such as the structure of AMPs. In future work, on the one hand, we plan to integrate information from multiple modalities such as AMP sequences, structures and interaction networks to improve the performance of each module as much as possible. On the other hand, it is planned to improve the performance of the entire Diff-AMP framework from the perspective of collaborative work.

**Key Points**

- Diff-AMP: integrated framework for efficient AMP generation, identification, prediction and optimization.
- RLGen: uses diffusion strategy for improved AMP generation via enhanced amino acid interdependence.
- Enhanced AMP recognition model developed with a high-parameter pre-training model for better node representation and screening efficiency.
- Reinforcement learning-based model for generating customized AMPs with specific attributes and lengths.

## Data Availability

Our data and code are publicly available at: https://github.com/wrab12/diff-amp. And online webserver is publicly accessed at: https://huggingface.co/spaces/jackrui/Diff-AMP.

## Author Biographies

**Rui Wang, Tao Wang, and Jinhang Wei** are engaged in studies at Wenzhou University of Technology, where **Linlin Zhuo** also contributes as a professional staff member. Their collective research endeavors are concentrated on the prediction of drug-target interactions, the assessment of molecular metabolic stability, and the innovative design of antimicrobial peptides, showcasing a broad and impactful range of interests within the scientific community.

**Xiangzheng Fu**, a postdoctoral fellow at Hunan University, specializes in bioinformatics.

**Quan Zou**, serving as a professor at the University of Electronic Science and Technology of China, also focuses on bioinformatics.

**Xiaojun Yao**, a professor at Macao Polytechnic University, dedicates his research to bioinformatics.
